# A randomized, double-blind, placebo-controlled trial of calcium acetate on serum phosphorus concentrations in patients with advanced non-dialysis-dependent chronic kidney disease

**DOI:** 10.1186/1471-2369-12-9

**Published:** 2011-02-16

**Authors:** Wajeh Qunibi, Wolfgang C Winkelmayer, Richard Solomon, Moustafa Moustafa, Paul Kessler, Chiang-Hong Ho, Jonathan Greenberg, Jose A Diaz-Buxo

**Affiliations:** 1 Department of Medicine, University of Texas Health Sciences Center, 7703 Floyd Curl Drive, San Antonio, TX, 78229, USA; 2 Division of Nephrology, Department of Medicine, Stanford University School of Medicine, 780 Welch Road, Suite 106, Palo Alto, CA 94304, USA; 3 Department of Medicine, 2308 Rehab 2, UHC Campus Fletcher Allen Health Care, University of Vermont, Burlington, VT, 05401, US; 4 South Carolina Nephrology and Hypertension, 1184 Orangeburg Mall Circle, Orangeburg, SC 29115-3439, USA; 5 Clinical, Medical and Regulatory Affairs, Nabi Biopharmaceuticals, 12276 Wilkins Avenue, Rockville, MD, 20852, USA; 6 Renal Therapies Group, Fresenius Medical Care, 309 East Morehead Street, Suite 285, Charlotte, NC, 28202, USA; 7 Daiichi Sanky, 399 Thornall Street, Edison, New Jersey 08837, USA

## Abstract

**Background:**

Hyperphosphatemia in patients with chronic kidney disease (CKD) contributes to secondary hyperparathyroidism, soft tissue calcification, and increased mortality risk. This trial was conducted to examine the efficacy and safety of calcium acetate in controlling serum phosphorus in pre-dialysis patients with CKD.

**Methods:**

In this randomized, double-blind, placebo-controlled trial, 110 nondialyzed patients from 34 sites with estimated GFR < 30 mL/min/1.73 m^2 ^and serum phosphorus > 4.5 mg/dL were randomized to calcium acetate or placebo for 12 weeks. The dose of study drugs was titrated to achieve target serum phosphorus of 2.7-4.5 mg/dL. Serum phosphorus, calcium, iPTH, bicarbonate and serum albumin were measured at baseline and every 2 weeks for the 12 week study period. The primary efficacy endpoint was serum phosphorus at 12 weeks. Secondary endpoints were to measure serum calcium and intact parathyroid hormone (iPTH) levels.

**Results:**

At 12 weeks, serum phosphorus concentration was significantly lower in the calcium acetate group compared to the placebo group (4.4 ± 1.2 mg/dL *vs*. 5.1 ± 1.4 mg/dL; *p *= 0.04). The albumin-adjusted serum calcium concentration was significantly higher (9.5 ± 0.8 vs. 8.8 ± 0.8; *p *< 0.001) and iPTH was significantly lower in the calcium acetate group compared to placebo (150 ± 157 vs. 351 ± 292 pg/mL respectively; *p *< 0.001). At 12 weeks, the proportions of subjects who had hypocalcemia were 5.4% and 19.5% for the calcium acetate and the placebo groups, respectively, while the proportions of those with hypercalcemia were 13.5% and 0%, respectively. Adverse events did not differ between the treatment groups.

**Conclusions:**

In CKD patients not yet on dialysis, calcium acetate was effective in reducing serum phosphorus and iPTH over a 12 week period.

**Trial Registration:**

www.clinicaltrials.gov NCT00211978.

## Background

Chronic kidney disease (CKD) is a major global health problem affecting an estimated 5 to 10 percent of the total world population [1]. In the United States, the prevalence of CKD has increased from 10% in 1988-1994 to 13.1% in 1999-2004 with an estimated 26 million individuals with CKD [2,3]. Of these, more than 8 million are estimated to have a glomerular filtration rate (GFR) < 60 mL/minute/1.73 m^2^. Chronic kidney disease is associated with increased cost and poor clinical outcomes [1-4]. In addition to the progressive loss of kidney function over time and the eventual need for renal replacement therapy, patients with CKD have a high mortality rate, with cardiovascular disease accounting for almost 50% of deaths [3]. In fact, patients with CKD are more likely to die than progress to end-stage renal disease (ESRD) [4]. Strong evidence from experimental and clinical studies has documented a major role of elevated serum phosphorus in the pathogenesis of several clinical disorders in patients with CKD [5-13]. Hyperphosphatemia contributes to abnormal bone metabolism and cardiovascular calcification (CVC); components of the syndrome of CKD-Mineral and Bone Disorder (CKD-MBD) [14]. Ample evidence now exists for the role of hyperphosphatemia in the pathogenesis of CVC, a risk factor for death from cardiovascular disease [15-21]. In vitro studies showed that phosphorus directly stimulates vascular smooth muscle cells to undergo osteoblastic differentiation and expression of bone-related proteins that are involved in the development and progression of CVC [16,18,20]. More important is the association between high serum phosphorus and increased risk of cardiovascular events and mortality, both in the CKD population [5,22] as well as in individuals with normal renal function [23]. On the other hand, use of phosphate binders decreased vascular calcification in mice [18]. Thus, control of serum phosphorus to levels recommended by KDOQI guidelines for bone and mineral metabolism during earlier stages of CKD may help reduce cardiovascular morbidity and mortality [24]. Indeed, treatment with phosphorus binders was recently shown to be independently associated with improved survival among incident hemodialysis patients [6]. If these findings can be substantiated, the potential benefits of phosphate binders may extend to the non-dialyzed CKD population. Due to the scarcity of high-level evidence on the beneficial effect of phosphate binders on hard study outcomes, however, none of the currently available phosphate binders have received approval by the U.S. Food and Drug Administration (FDA) for use in patients with CKD who do not require dialysis.

Calcium acetate is an effective phosphate binder that is routinely used in patients with ESRD [25-27]. The aim of the present randomized, double-blind, placebo-controlled trial was to evaluate the efficacy and safety of calcium acetate in controlling serum phosphorus, calcium, PTH, and Ca × P product in nondialyzed patients with stage 4 and 5 CKD.

## Methods

### Study population

The target population was patients with advanced CKD who did not yet require dialysis and whose serum phosphorus was elevated in the absence of phosphate binder therapy. We hypothesized that calcium acetate would safely improve such patients' phosphorus control compared with placebo.

### Study Design

This study was a prospective, multicenter, randomized, double-blind, placebo-controlled efficacy trial to compare the effect of calcium acetate versus placebo on the control of serum phosphorus, iPTH, and the calcium-phosphorus product in hyperphosphatemic, pre-dialysis patients with advanced CKD as defined by a GFR < 30 mL/min/1.73 m^2^. The study protocol and the informed consent form were reviewed and approved by the Institutional Review Boards (IRB) of each participating center and registered at www.clinicaltrials.gov (NCT00211978.)

Study participants had to be at least 18 years of age, have an estimated GFR < 30 mL/min/1.73 m^2 ^as estimated by the four variable Modification of Diet in Renal Disease (MDRD) equation, and a serum phosphorus > 4.5 mg/mL following a washout period of 6 weeks. During the washout period, all phosphate binders, calcium supplements, and vitamin D analogues were discontinued if previously taken. Patients were excluded if they had history of medications non-adherence, gastrointestinal motility disorders, or any other conditions that rendered them clinically unstable. All patients signed written, informed consent prior to the initiation of any study-related activities.

Patients were instructed to continue their usual diet. The starting dose of study drug was guided by the serum phosphorus level at the end of the washout period. Patients with serum phosphorus levels between 4.5 and 5.0 mg/dL received an initial dose of 1 gelcap per meal; those with phosphorus levels between 5.1 and 6.0 mg/dL started with 2 gelcaps per meal and those with phosphorus levels > 6.0 mg/dL were administered a starting dose of 3 gelcaps per meal. Study participants returned for follow-up visits every 2 weeks. During these visits, the dose was titrated up to a maximum of 15 gelcaps per day. If, after 3 months of treatment, the serum phosphorus level remained > 5.5 mg/dL or the iPTH was still > 110 pg/mL despite maximum daily dose of 15 gelcaps, the study protocol required that such subjects be withdrawn from the study for failure to control. The use of medications that alter calcium and phosphorus balance was prohibited throughout the study. Thus, the use of rescue medications such as other phosphate binders, calcium supplements or vitamin D was also considered a study failure.

Serum phosphorus, calcium, iPTH, bicarbonate and serum albumin were measured at baseline and every 2 weeks throughout the study period. Laboratory samples were centrally analyzed by LabConnect, Seattle, WA. Total calcium was corrected for serum albumin. Calcium and phosphorus were measured as colorimetric assays on the Olympus 5400 analyzer. Intact PTH levels and homocysteine were assayed by a chemiluminescent assay on the Bayer Advia Centaur.

For the purpose of this study, the following recommended KDOQI treatment targets were used to categorize patients as being in target, below, or above target: serum phosphorus of 2.7 to 4.5 mg/dL, and iPTH of 70 to 110 pg/mL. Target serum calcium was defined as 8.5 to 10.2 mg/dL adjusted for serum albumin. Patients were also assessed for compliance with study medications, adverse events, and use of concomitant medications during each study visit.

### Statistical Analysis

All analyses followed the intention-to-treat (ITT) principle. The original study plan called for 26 weeks of follow-up with the proportion of weeks within target being the efficacy measure (see Additional file 1: A for the original sample-size calculation). As a consequence of the requirement that participants with uncontrolled hyperphosphatemia be removed from the study after 3 months, a large proportion of patients in the placebo group left the study at that time point. Thus, by such design, informative censoring was introduced into the study. The present analysis departs from this study plan and limits observation to 12 weeks from randomization to reduce such bias. The primary efficacy measure was the serum phosphorus at 12 weeks. Secondary endpoints included serum calcium adjusted for serum albumin and iPTH levels at 12 weeks.

Baseline characteristics were compared using the 2-sample t-test and Pearson's chi-square tests for continuous and categorical variables, respectively. Measurements at 12 weeks were also compared using the 2-sample t-test. All statistical tests were two-sided with a p-value of < 0.05 considered significant. In addition to the comparison of the biochemical measurements at 12 weeks, the parameters were also assessed throughout the 12-week observation period, together with the corresponding standard deviations. All analyses were performed using SAS release 9.1.

## Results

**Figure 1:**
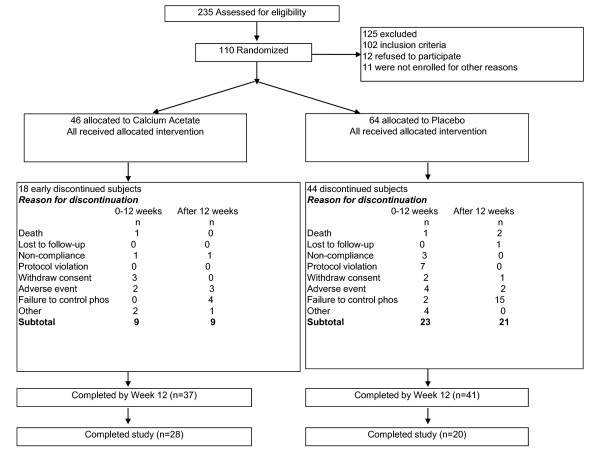
**Patient disposition from screening to end of study**.

A total of 235 patients were screened and 110 were randomly allocated to receive calcium acetate (PhosLo^® ^667 mg capsules; Fresenius Medical Care North America, Waltham, MA, USA) or placebo for 6 months. Figure 1 provides a flow diagram of subjects from screening to completion of the study. The most common reason for screening failure was a serum phosphorus level below 4.5 mg/dL. Forty six subjects were treated with calcium acetate and 64 with placebo. Randomization errors in the use of block randomization at 3 study sites as well as randomization of small numbers of patients per study site resulted in more subjects randomized to placebo which contributed to the imbalance. Table 1 demonstrates, however, that randomization achieved balance of observed characteristics between treatment groups. Of the enrolled subjects, 9 (19.6%) subjects in the calcium acetate group and 23 (39.1%) in the placebo group dropped out of the study during the first 12 weeks (*p *= 0.06). As shown in Figure 1, one reason for dropping out was failure to control serum phosphorus, which was the reason for withdrawal in 0 and 2 patients in the calcium acetate and placebo groups, respectively. Based on pill counts obtained at each visit, compliance during the 12 week study period was similar in the two groups: 88.6 ± 15.0% in the calcium acetate group and 89.3 ± 14.0% in the placebo group. Two patients in each group were withdrawn from the study because they started dialysis.

**Table 1:** Baseline Characteristics

Characteristic	Calcium acetate (N = 46)	Placebo (N = 64)	P-value
Age (years)	63.2 ± 11.7	62.2 ± 14.2	0.69

Gender (female)	23 (50%)	29 (45.3%)	0.63

Race (white)	33 (71.7%)	54 (84.4%)	0.34

Diabetes	31 (67.4%)	49 (76.6%)	0.29

Weight (Kg)	82.9 ± 23.2	80.8 ± 25.6	0.65

Serum phosphorus (mg/dL)	5.1 ± 1.2	5.1 ± 1.1	0.76

Serum calcium, corrected (mg/dL)	9.1 ± 0.7	9.1 ± 0.6	0.97

			

Intact parathyroid hormone (pg/mL)^1^	253 ± 213	322 ± 267	0.16

Serum albumin (mg/dL)	3.6 ± 0.5	3.5 ± 0.5	0.56

Serum bicarbonate (mEq/L)	22.6 ± 3.9	21.9 ± 3.7	0.33

Estimated glomerular filtration rate (mL/min/1.73 m^2^)	17.3 ± 5.6	16.4 ± 6.2	0.43

^1^Available in 45 and 56 patients in the calcium acetate and placebo groups, respectively.

### Serum phosphorus concentration

**Figure 2:**
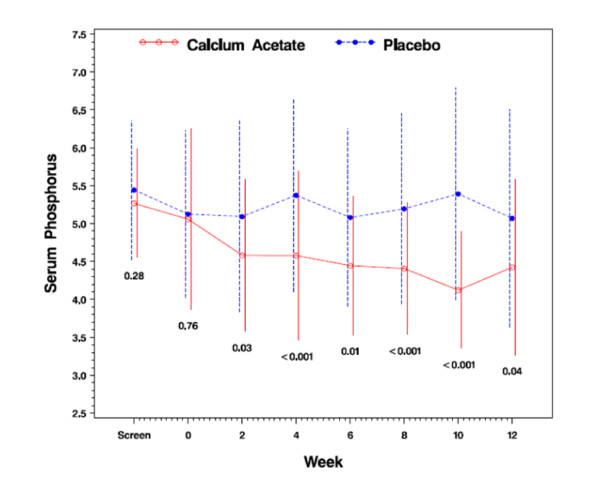
**Serum phosphorus concentration (mg/dL) from randomization to 12 weeks**.

**Figure 3:**
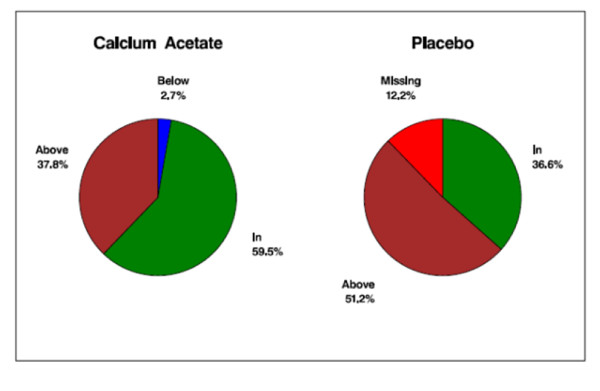
**Proportion (%) of patients in, below, or above the target range of serum phosphorus concentration (2.7-4.5 mg/dL) at 12 weeks**.

Baseline serum phosphorus concentration did not differ between treatment groups (5.1 ± 1.2 mg/dL *vs*. 5.1 ± 1.1 mg/dL in calcium acetate and placebo groups, respectively; *p *= 0.76; Table 1). Serum phosphorus concentration at 12 weeks, the primary efficacy endpoint, was significantly lower in the calcium acetate group compared to the placebo group (4.4 ± 1.2 mg/dL *vs*. 5.1 ± 1.4 mg/dL; *p *= 0.04; Table 2). Figure 2 displays the time course of serum phosphorus concentrations over the 12 weeks of investigation. The percentage of subjects with serum phosphorus control to the KDOQI target was significantly higher in the calcium acetate group compared with the placebo group (59.5% vs. 36.6%; *p *= 0.04; Figure 3).

**Table 2:** Comparison of study endpoints at 12 weeks by randomized treatment assignment

Characteristic	Calcium acetate (N = 37)	Placebo (N = 41)	P-value
Serum phosphorus (mg/dL)	n = 37 4.4 ± 1.2	n = 36 5.1 ± 1.4	0.04

Serum calcium, corrected (mg/dL)	n = 37 9.5 ± 0.8	n = 36 8.8 ± 0.8	< 0.001

Intact parathyroid hormone (pg/mL)	n = 35 150 ± 157	n = 38 351 ± 292	< 0.001

Serum bicarbonate (mEq/L)	n = 35 24.0 ± 3.7	n = 35 21.6 ± 3.8	0.008

Serum albumin (mg/dL)	n = 37 3.6 ± 0.5	n = 36 3.5 ± 0.5	0.34

### Serum calcium concentration

**Figure 4:**
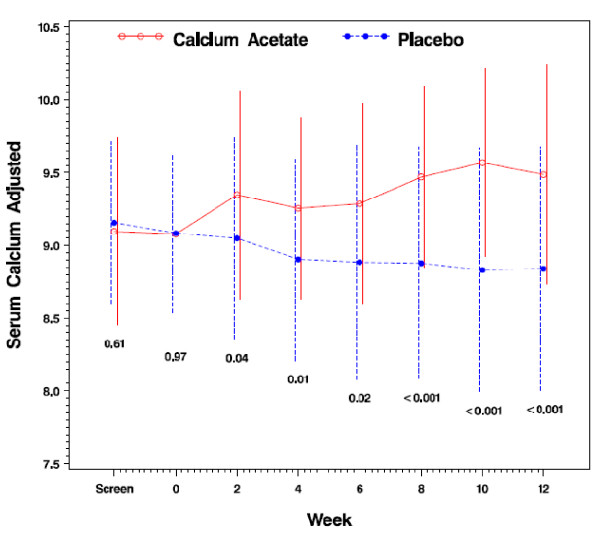
**Serum calcium concentration (mg/dL) from randomization to 12 weeks**.

**Figure 5:**
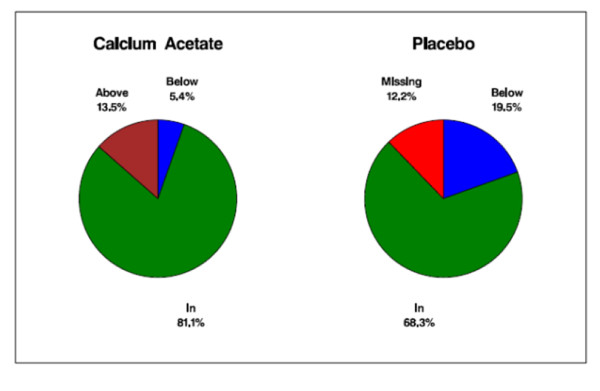
**Proportion (%) of patients in, below, or above the target range of serum calcium concentration (8.5-10.2 mg/dL) at 12 weeks**.

Serum calcium concentrations, adjusted for serum albumin, did not differ between the randomized groups at baseline (9.1 ± 0.7 vs. 9.1 ± 0.6; *p *= 0.97; Table 1). After 12 weeks of study, subjects in the calcium acetate group had significantly higher albumin-adjusted serum calcium concentrations compared with the placebo group (9.5 ± 0.8 vs. 8.8 ± 0.8; *p *< 0.001; Table 2) (Figure 4). The proportion of subjects within the target range, however, did not differ between treatment groups (81.1% vs. 71.8%; *p *= 0.34; Figure 5). Most subjects outside the target range in the calcium acetate group were above the upper limit (5 patients; 13.5% of total), whereas no placebo treated subject was above the targeted range in the placebo group. At 12 weeks, the percentage of subjects who had hypocalcemia was 5.4% and 19.5% for the calcium acetate and the placebo groups respectively while the proportions of those with hypercalcemia were 13.5% and 0%, respectively. There were 18 episodes of hypercalcemia in the calcium acetate group and 5 in the placebo group.

### Intact parathyroid hormone concentrations (iPTH)

**Figure 6:**
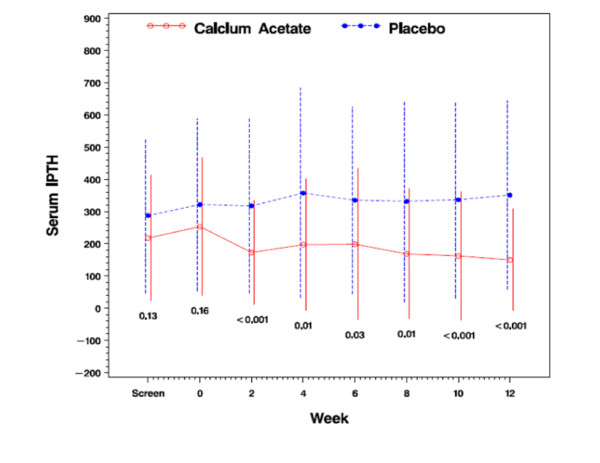
**Serum intact parathyroid hormone concentration (pg/mL)**.

**Figure 7:**
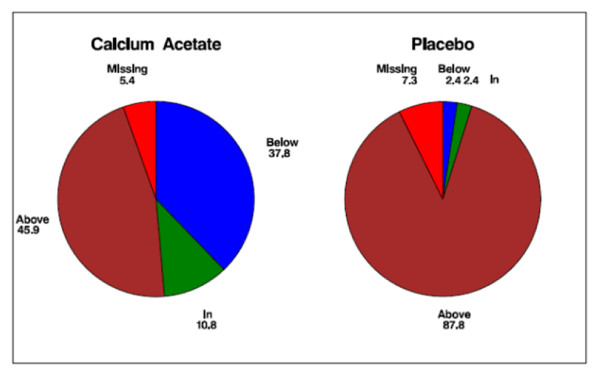
**Proportion (%) of patients in, below, or above the target range of serum parathyroid hormone level (70-110 pg/mL) at 12 weeks**.

Serum concentrations of iPTH did not differ between treatment groups at baseline (Table 1). At 12 weeks, however, iPTH was significantly lower in the calcium acetate group compared with placebo (150 ± 157 vs. 351 ± 292 pg/mL; *p *< 0.001; Figure 6). Suppression of iPTH in the treatment group was apparent after 2 weeks of treatment (Figure 6). While only 45.9% of subjects in the calcium acetate group had an iPTH concentration above 110 pg/dL after 12 weeks, almost all subjects had elevated iPTH in the placebo group at that time point (92.3%; *p *< 0.001; Figure 7).

### Safety and tolerability

During 12 weeks of follow up, 65.2% of subjects in the calcium acetate and 65.6% of subjects in the placebo group reported at least one adverse event (*p *= 0.96; Table 3). The rates of adverse events in general did not differ between study groups: 0.42 versus 0.57 events per subject per visit in the calcium acetate and placebo groups, respectively (*p *= 0.46). Adverse events deemed related to the study drug, were relatively rare: 10.9% and 14.1% of patients in the calcium acetate and placebo groups experienced at least 1 event (*p *= 0.16). The rate of related adverse events also did not differ between study groups (*p *= 0.84). Additional file 1: B displays all adverse events and related adverse events in each study group in full detail

**Table 3:** Adverse events prior to week 14

Adverse Event Outcome	Calcium acetate (N = 46)	Placebo (N = 64)	P-value
Patients with any adverse event	30 (65.2%)	42 (65.6%)	0.96

Any adverse event, rate (per subject per visit)	0.42	0.57	0.46

Patients with related adverse event	5 (10.9%)	9 (14.1%)	0.16

Related adverse event, rate (per subject per visit)	0.39	0.36	0.84

Adverse events related to medication by organ system class			

Cardiac disorders	0	1 (1.6%)	

Gastrointestinal disorders	3 (6.5%)	6 (9.4%)	

General disorders	1 (2.2%)	0	

Investigations	1 (2.2%)	0	

Metabolism and nutrition disorders	2 (4.3%)	1 (1.6%)	

Musculoskeletal and connective tissue disorders	0	1 (1.6%)	

Skin and subcutaneous tissue disorders	0	1 (1.6%)	

Vascular disorders	0	1 (1.6%)	

## Discussion

KDOQI guidelines recommend that serum phosphorus be maintained within the target range of 2.7 to 4.6 mg/dL in stages 3 and 4 CKD and 3.5 to 5.5 mg/dL in stage 5 CKD by means of dietary phosphate restriction, dialysis and use of phosphate binders [24]. In this study, calcium acetate resulted in reduction of the mean serum phosphorus to recommended levels in pre-dialysis patients with advanced CKD compared with placebo (4.4 ± 1.2 mg/dL *vs*. 5.1 ± 1.4 mg/dL, respectively; *p *= 0.04). The percentage of subjects whose serum phosphorus concentration fell within target was significantly higher in the calcium acetate group compared with placebo (59.5% vs. 36.6% p = 0.04; Figure 3).

Phosphate binders are approved for the treatment of hyperphosphatemia in patients receiving maintenance dialysis based on their efficacy on the endpoint of phosphorus and possibly PTH control. Their efficacy in reducing relevant endpoint such as morbidity or mortality has never been established. One study has demonstrated a beneficial association between receipt of phosphorus binder (calcium-based or non-calcium based), but it cannot be ruled out that patients receiving phosphate binders were systematically healthier [6]. Given that CKD is a clinical continuum and that a serum phosphorus level greater than 3.5 mg/dL is an independent predictor of all-cause mortality in dialyzed and non-dialyzed patients [5,22,23], it is also possible that controlling elevated serum phosphorus concentrations may yield beneficial results, but this remains to be shown in randomized trials. None of the currently available phosphate binders are approved by the FDA for use in pre-dialysis patients. The present study is the first to provide unequivocal evidence from a randomized trial that calcium acetate is efficacious in reducing serum phosphorus concentrations in patients with predialysis CKD. As a phosphate binder, calcium acetate offers a number of potential advantages in non-dialyzed CKD patients. *First*, as shown in this trial, calcium acetate is clearly effective in controlling serum phosphorus to levels recommended by KDOQI guidelines. One could assume that achieving these targets in CKD patients is associated with a reduction in the risk of developing cardiovascular calcification and bone disease and a reduction in the risk of death. Treatment with any of the available phosphate binders was shown to be independently associated with improved survival among incident hemodialysis patients [6]. In addition, calcium acetate can correct hypocalcemia which is a critical factor in stimulating PTH secretion and induction of parathyroid gland hyperplasia. During this study, hypocalcemia was observed in 5.4% and 19.5% for the calcium acetate and the placebo groups respectively. On the other hand, there was a small but significant increase in serum calcium in the calcium acetate group rendering it more effective than placebo in suppressing PTH secretion (Table 2 and Figure 6). Calcium acetate has a salutary effect on acidosis which is known to contribute to the pathogenesis of renal bone disease, malnutrition and inflammation [28,29]. The acetate anion is metabolized to bicarbonate which helps to correct metabolic acidosis, a common abnormality in non-dialyzed CKD patients [29]. The serum bicarbonate level at the end of our study was significantly higher in calcium acetate treated subjects than in those in the placebo group (24.0 ± 3.7 vs. 21.6 ± 3.8 mEq/L respectively; p-value 0.008).

Calcium acetate was well tolerated and exhibited a safety profile similar to placebo. The most frequent adverse events in calcium acetate-treated subjects were gastrointestinal events as well as "infections and infestations", both not significantly different from those recorded in the placebo group.

Few studies have examined the efficacy and safety of phosphate binders in non-dialyzed CKD patients [30-32]. Borrego and colleagues compared the efficacy of calcium carbonate vs. calcium acetate as phosphate binders in 28 patients with CKD (mean creatinine clearance 21 ml/min) [30]. These authors found that both drugs were similarly effective as phosphate binders in lowering serum phosphate levels. The carbonate group, though, required a four-fold greater dose of calcium than the acetate group and exhibited more hypercalcemic episodes [30]. In another study, Ketteler et al showed that sevelamer carbonate is effective in controlling serum phosphorus in CKD patients [31]. However, their study design was an open-label, single-arm 8 week study while our study was a randomized, double-blind, and placebo-controlled 12 week study. Moreover, subjects in their study were required to have serum phosphate level of ≥ 5.5 mg/dl for enrollment in contrast to the > 4.5 mg/dl in our study. Finally, the iPTH level was reduced to a lower level in our study, obviating the need for administration of other drugs that suppress PTH secretion. Conversely, Ketteler and colleagues prescribed a daily dose of 400 IU of the native form of vitamin D during the treatment period and several subjects in their study were continued on pre-study 1,25 dihydroxyvitamin D and/or cinacalcet. A more recent study by Sprague et al reported the efficacy and safety of lanthanum carbonate in 121 patients with CKD stages 3 and 4 for 8 weeks with 90 patients completing the study [30]. Their study and ours were similar in that both were randomized, double-blind, placebo-controlled trials. However, our study was for slightly longer duration (12 weeks vs. 8 weeks) and a higher percentage of out calcium acetate-treated subjects achieved serum phosphorus levels within target compared to their study subjects (59.5% versus 44.6% respectively).

A major limitation of our study is the drop-out rate and withdrawal rate for failure to control serum phosphorus or iPTH levels. In order to address the impact of the dropouts, an ITT analysis was used where all patients who received at least one dose of the study drug were included. This conservative analysis which was based on all randomized subjects may have lowered the percentage of those who achieved target serum phosphorus and iPTH levels in calcium acetate-treated patients. Urinary excretion of phosphorus was not measured in this trial. Previous studies have shown that administration of phosphate binders resulted in a significant decrease in urinary phosphorus excretion in CKD patients [30,31]. Finally, the present study did not examine patients' outcomes as this usually requires a much larger sample size. However, a previous study has shown that high serum phosphorus concentrations in patients with CKD was associated with significantly greater progression of coronary artery calcification (CAC) even in patients whose serum phosphorus was within the normal range [21]. Treatment with phosphate binders stabilized or even decreased progression of CAC in these patients [33]. However, although serum calcium levels might be normal, it is possible to develop a positive calcium balance when high doses of calcium based binders are used. Thus, further investigations are warranted in order to assess the long-term effects of chronic treatment with calcium-based or non-calcium containing in patients with non-dialysis-dependent CKD.

## Conclusion

The results of this study demonstrated that calcium acetate is efficacious and safe in controlling serum phosphorus, calcium, iPTH and serum bicarbonate in non-dialyzed CKD patients. Additional studies are clearly required, not only to study the efficacy and safety of phosphate binders in CKD patients, but also to investigate their impact on patient outcomes.

## Competing interests

Dr. Qunibi is a consultant for Fresenius Medical Care, served on advisory boards for Fresenius Medical Care and received research support from Fresenius. Dr. Winkelmayer served as an advisor and consultant to Fresenius Medical Care. Dr. Kessler is an employee of Nabi Biopharmaceuticals. Dr. Greenberg was an employee with Fresenius Medical Care and is now with Daiichi Sankyo. Dr. Diaz-Buxo is an employee of Fresenius Medical Care. The remaining authors received research support from Fresenius Medical Care.

The following investigators have participated in the EPICK study.

Wajeh Qunibi, MD. San, Antonio, TX

Moustafa Moustafa, Orangeburg, SC

Alexander Ackad, MD. Hackensack, NJ

Corey Anderson, MD. Sun City, AZ

Michael Austerlitz, MD. Los Angeles, CA

Marializa Bernardo, MD. Houston, TX

Vito Campese, MD. Covina, CA

Wei-Tzuoh Chen, MD. Visalia, CA

Roderick Clark, MD. Lafayette, LA

R. Michael Culpepper, MD. Mobile, AL

Hem Deodhar, MD. Portland, OR

Francis Dumler, MD. Royal Oak, MI

Eben Feinstein, MD. Los Angeles, CA

Francis Foti, MD. Erie, PA

Roger Haley, MD. Visalia, CA

Steven Hays, MD. Dallas, TX

Joachim Hertel, MD. Augusta, GA

Fred Husserl, MD. New Orleans, LA

Michel Jadoul. Brussels, Belgium

Golriz Jafari, MD. Montebello, CA

Joseph Krause, MD. Delray Beach, FL

Ira Lazar, MD. Boca Raton, FL

Baudouin Leclerq, MD. Ocala, FL

James Lewis, III, MD. Birmingham, AL

Y. Howard Lien, MD, PhD. Tucson, AZ

Jeffrey Martin, MD. Lancaster, PA

Carlos Martinez, MD. Macon, GA

Tarun Marwaha, MD. Fountain Valley, CA

Peter McCauley, MD. Bakersfield, CA

Kevin McConnell, MD. Charlottesville, VA

Ramon Mendez, MD. Alexandria, VA

Neville Pokroy, MD. Las Vegas, NV

Thomas Rakowski, MD. Fairfax, VA

John Robertson, MD. Riverside, CA

Jack Rubin, MD. Los Alamitos, CA

Mark Russo, MD. Naples, FL

David Scott, MD. Springfield Gardens, NY

Mohamed Sekkarie, MD. Bluefield,WV

Gerard Sigue, MD. Lafayette, LA

Bhupinder Singh, MD. Tempe, AZ

Harmeet Singh, MD. Lakewood, CO

Richard Solomon, MD. Burlington, VT

Amy Sprague, MD. Augusta, GA

Steven Steinberg MD. San Diego, CA

Pusadee Suchinda, MD. Sumter, SC

Ariv Swaminathan, MD. Phoenix, AZ

Nirupama Vemuri, MD. Coral Springs, FL

Dierik Verbeelen. Brussels, Belgium

Melchiore Vernace, MD. Doylestown, PA

Timothy Youell, MD. Orlando, FL

Raja Zabaneh, MD. Shreveport, LA

## Authors' contributions

WQ wrote the manuscript. WQ, RS, and MM contributed patients to the study. WCW and C-H H provided statistical analysis. PK contributed to the design of study protocol and over saw conduct of the study. JG and JAD-B helped edit the manuscript. All authors have read and approved the final manuscript.

## Acknowledgements

**Grant Support: **This study was supported by a grant from Fresenius Medical Care North America, Waltham, MA. The study was presented in part at the annual meeting of the National Kidney Foundation in Orlando, FL, April 2007. We would like to acknowledge the contributions of patients who participated in the study; all study coordinators for their help with patient recruitment and conduct of the study and the staff of the renal clinics who were all helpful in many ways.

## Pre-publication history

The pre-publication history for this paper can be accessed here:

http://www.biomedcentral.com/1471-2369/12/9/prepub

## Supplementary Material

Additional file 1 **A. Sample size calculation in the original study protocol**. File contains calculation of the sample size. B. Adverse Events (AE) in the calcium acetate and placebo groups. File contains all adverse events in the study subjects. Click here for file
