# Supplementary material for: A randomized, double-blind, placebo-controlled trial of calcium acetate on serum phosphorus concentrations in patients with advanced non-dialysis-dependent chronic kidney disease
Source: BMC Nephrol. 2011 Feb 16;12:9. doi: 10.1186/1471-2369-12-9 (PMC3055808; doi:10.1186/1471-2369-12-9)
Supplement: Additional file 1 — A. Sample size calculation in the original study protocol. File contains calculation of the sample size. B. Adverse Events (AE) in the calcium acetate and placebo groups. File contains all adverse events in the study subjects. [file 1471-2369-12-9-S1.DOCX]

**Additional file A - Sample size calculation in the original study protocol**

Under the assumptions that subjects in the calcium acetate group would be on-target in 50% of the 24-week study period *vs*. 10% in the placebo group, and 50% as the standard deviation of the proportion of weeks on-target, 34 patients per group would yield 90% power for a two-sided test with 5% type I error rate. With a symmetrical 20% drop-out rate in each group, 43 patients per group for a total of 86 patients were required.

**Additional file B – Adverse Events (AE) in the calcium acetate and placebo groups**

Fresenius Medical Care - North America 1 of 7

EPICK

Appendix B Table 1

Adverse Events prior to Day 98 Visit - ITT population

_____________________________________________________________________________________________________________________________________

_______PhosLo________ _______Placebo_______

Subject Event Subject Event

(N=46) (N=64)

System Organ Class n(%) n n(%) n

Preferred Term p-val

_____________________________________________________________________________________________________________________________________

At Least One AE 30 (65.2) 42 (65.6) 0.9646

BLOOD AND LYMPHATIC SYSTEM DISORDERS 3 ( 4.7) 3

ANAEMIA 2 ( 3.1) 2

MICROCYTIC ANAEMIA 1 ( 1.6) 1

CARDIAC DISORDERS 3 ( 6.5) 3 5 ( 7.8) 7

ANGINA PECTORIS 1 ( 1.6) 1

ARTERIOSCLEROSIS CORONARY ARTERY 1 ( 2.2) 1

CARDIAC FAILURE CONGESTIVE 2 ( 4.3) 2 2 ( 3.1) 2

DILATATION ATRIAL 1 ( 1.6) 1

PALPITATIONS 1 ( 1.6) 1

TACHYCARDIA 1 ( 1.6) 1

VENTRICULAR HYPERTROPHY 1 ( 1.6) 1

ENDOCRINE DISORDERS 1 ( 2.2) 1 2 ( 3.1) 2

HYPERPARATHYROIDISM 2 ( 3.1) 2

HYPERTHYROIDISM 1 ( 2.2) 1

EYE DISORDERS 1 ( 2.2) 1

CONJUNCTIVITIS 1 ( 2.2) 1

GASTROINTESTINAL DISORDERS 11 (23.9) 17 19 (29.7) 31

ABDOMINAL HERNIA 1 ( 1.6) 1

ABDOMINAL PAIN 1 ( 2.2) 1 2 ( 3.1) 2

_____________________________________________________________________________________________________________________________________

ITT population included all subjects who received at least one dose of the study medication.

Blanks represented no adverse events recorded.

The p-values was derived using Pearson''s Chi-square test.

Data Version Date: 25SEP2008 t4.1.sas executed at 24JUL2009 13:04

Fresenius Medical Care - North America 2 of 7

EPICK

Appendix B Table 1

Adverse Events prior to Day 98 Visit - ITT population

_____________________________________________________________________________________________________________________________________

_______PhosLo________ _______Placebo_______

Subject Event Subject Event

(N=46) (N=64)

System Organ Class n(%) n n(%) n

Preferred Term p-val

_____________________________________________________________________________________________________________________________________

ABDOMINAL PAIN UPPER 1 ( 2.2) 2 1 ( 1.6) 1

CONSTIPATION 1 ( 2.2) 1 2 ( 3.1) 2

DIABETIC GASTROPARESIS 1 ( 2.2) 1

DIARRHOEA 4 ( 8.7) 5 5 ( 7.8) 5

DRY MOUTH 1 ( 1.6) 1

DYSPEPSIA 1 ( 2.2) 1 1 ( 1.6) 1

FLATULENCE 1 ( 1.6) 1

FREQUENT BOWEL MOVEMENTS 1 ( 2.2) 1

GASTROOESOPHAGEAL REFLUX DISEASE 4 ( 6.3) 5

HAEMATEMESIS 1 ( 1.6) 1

NAUSEA 2 ( 4.3) 3 7 (10.9) 8

VOMITING 2 ( 4.3) 2 3 ( 4.7) 3

GENERAL DISORDERS AND ADMINISTRATION SITE CONDITIONS 3 ( 6.5) 5 9 (14.1) 15

CHEST DISCOMFORT 1 ( 1.6) 1

FATIGUE 1 ( 2.2) 1 2 ( 3.1) 2

FEELING HOT 1 ( 2.2) 1

GENERALISED OEDEMA 1 ( 1.6) 1

INFLUENZA LIKE ILLNESS 1 ( 1.6) 1

MALAISE 1 ( 2.2) 1 1 ( 1.6) 1

OEDEMA 1 ( 1.6) 1

_____________________________________________________________________________________________________________________________________

ITT population included all subjects who received at least one dose of the study medication.

Blanks represented no adverse events recorded.

The p-values was derived using Pearson''s Chi-square test.

Data Version Date: 25SEP2008 t4.1.sas executed at 24JUL2009 13:04

Fresenius Medical Care - North America 3 of 7

EPICK

Appendix B Table 1

Adverse Events prior to Day 98 Visit - ITT population

_____________________________________________________________________________________________________________________________________

_______PhosLo________ _______Placebo_______

Subject Event Subject Event

(N=46) (N=64)

System Organ Class n(%) n n(%) n

Preferred Term p-val

_____________________________________________________________________________________________________________________________________

OEDEMA PERIPHERAL 1 ( 2.2) 2 4 ( 6.3) 7

PAIN 1 ( 1.6) 1

HEPATOBILIARY DISORDERS 1 ( 2.2) 1

BILIARY DYSKINESIA 1 ( 2.2) 1

INFECTIONS AND INFESTATIONS 9 (19.6) 10 9 (14.1) 10

ARTERIOVENOUS GRAFT SITE INFECTION 1 ( 1.6) 1

GASTROENTERITIS VIRAL 3 ( 6.5) 3

HERPES SIMPLEX 1 ( 2.2) 1

INFLUENZA 3 ( 4.7) 3

NASOPHARYNGITIS 1 ( 2.2) 1 1 ( 1.6) 1

PNEUMONIA 2 ( 4.3) 2 1 ( 1.6) 1

PNEUMONIA STAPHYLOCOCCAL 1 ( 1.6) 1

UPPER RESPIRATORY TRACT INFECTION 1 ( 2.2) 1 2 ( 3.1) 2

URINARY TRACT INFECTION 2 ( 4.3) 2

WOUND INFECTION 1 ( 1.6) 1

INJURY, POISONING AND PROCEDURAL COMPLICATIONS 1 ( 2.2) 1 4 ( 6.3) 4

ARTERIOVENOUS FISTULA SITE COMPLICATION 2 ( 3.1) 2

PROCEDURAL PAIN 1 ( 1.6) 1

SKIN LACERATION 1 ( 2.2) 1

VASCULAR GRAFT COMPLICATION 1 ( 1.6) 1

_____________________________________________________________________________________________________________________________________

ITT population included all subjects who received at least one dose of the study medication.

Blanks represented no adverse events recorded.

The p-values was derived using Pearson''s Chi-square test.

Data Version Date: 25SEP2008 t4.1.sas executed at 24JUL2009 13:04

Fresenius Medical Care - North America 4 of 7

EPICK

Appendix B Table 1

Adverse Events prior to Day 98 Visit - ITT population

_____________________________________________________________________________________________________________________________________

_______PhosLo________ _______Placebo_______

Subject Event Subject Event

(N=46) (N=64)

System Organ Class n(%) n n(%) n

Preferred Term p-val

_____________________________________________________________________________________________________________________________________

INVESTIGATIONS 2 ( 4.3) 3 3 ( 4.7) 5

BLOOD CALCIUM INCREASED 1 ( 2.2) 2

BLOOD GLUCOSE DECREASED 1 ( 2.2) 1

BLOOD PARATHYROID HORMONE INCREASED 1 ( 1.6) 1

BLOOD PHOSPHORUS INCREASED 1 ( 1.6) 1

BLOOD PRESSURE INCREASED 1 ( 1.6) 1

CARBON DIOXIDE ABNORMAL 1 ( 1.6) 1

PROTEIN URINE PRESENT 1 ( 1.6) 1

METABOLISM AND NUTRITION DISORDERS 8 (17.4) 8 7 (10.9) 13

ACIDOSIS 1 ( 1.6) 1

ANOREXIA 1 ( 2.2) 1 1 ( 1.6) 1

APPETITE DISORDER 1 ( 2.2) 1

DECREASED APPETITE 2 ( 3.1) 2

DEHYDRATION 1 ( 2.2) 1 1 ( 1.6) 1

FLUID OVERLOAD 1 ( 2.2) 1

HYPERCHOLESTEROLAEMIA 1 ( 2.2) 1

HYPERKALAEMIA 1 ( 2.2) 1 3 ( 4.7) 4

HYPERPHOSPHATAEMIA 1 ( 1.6) 1

HYPERURICAEMIA 1 ( 2.2) 1

HYPOCALCAEMIA 1 ( 2.2) 1 1 ( 1.6) 1

_____________________________________________________________________________________________________________________________________

ITT population included all subjects who received at least one dose of the study medication.

Blanks represented no adverse events recorded.

The p-values was derived using Pearson''s Chi-square test.

Data Version Date: 25SEP2008 t4.1.sas executed at 24JUL2009 13:04

Fresenius Medical Care - North America 5 of 7

EPICK

Appendix B Table 1

Adverse Events prior to Day 98 Visit - ITT population

_____________________________________________________________________________________________________________________________________

_______PhosLo________ _______Placebo_______

Subject Event Subject Event

(N=46) (N=64)

System Organ Class n(%) n n(%) n

Preferred Term p-val

_____________________________________________________________________________________________________________________________________

HYPOGLYCAEMIA 1 ( 1.6) 2

MUSCULOSKELETAL AND CONNECTIVE TISSUE DISORDERS 3 ( 6.5) 3 6 ( 9.4) 6

ARTHRALGIA 2 ( 3.1) 2

FISTULA 1 ( 1.6) 1

MUSCLE SPASMS 2 ( 3.1) 2

MYALGIA 1 ( 2.2) 1

OSTEOPOROSIS 1 ( 2.2) 1

PAIN IN EXTREMITY 1 ( 2.2) 1 1 ( 1.6) 1

NEOPLASMS BENIGN, MALIGNANT AND UNSPECIFIED 1 ( 2.2) 1

BASAL CELL CARCINOMA 1 ( 2.2) 1

NERVOUS SYSTEM DISORDERS 7 (15.2) 7 5 ( 7.8) 5

DIZZINESS 2 ( 4.3) 2 1 ( 1.6) 1

DYSARTHRIA 1 ( 2.2) 1

HEADACHE 2 ( 4.3) 2 2 ( 3.1) 2

HYPOAESTHESIA 1 ( 2.2) 1

LACUNAR INFARCTION 1 ( 2.2) 1

SOMNOLENCE 2 ( 3.1) 2

PSYCHIATRIC DISORDERS 2 ( 4.3) 2 2 ( 3.1) 3

ANXIETY DISORDER 1 ( 2.2) 1

CONFUSIONAL STATE 1 ( 2.2) 1

_____________________________________________________________________________________________________________________________________

ITT population included all subjects who received at least one dose of the study medication.

Blanks represented no adverse events recorded.

The p-values was derived using Pearson''s Chi-square test.

Data Version Date: 25SEP2008 t4.1.sas executed at 24JUL2009 13:04

Fresenius Medical Care - North America 6 of 7

EPICK

Appendix B Table 1

Adverse Events prior to Day 98 Visit - ITT population

_____________________________________________________________________________________________________________________________________

_______PhosLo________ _______Placebo_______

Subject Event Subject Event

(N=46) (N=64)

System Organ Class n(%) n n(%) n

Preferred Term p-val

_____________________________________________________________________________________________________________________________________

DEPRESSION 1 ( 1.6) 1

INSOMNIA 2 ( 3.1) 2

RENAL AND URINARY DISORDERS 2 ( 4.3) 2 2 ( 3.1) 3

AZOTAEMIA 1 ( 1.6) 1

RENAL FAILURE ACUTE 2 ( 4.3) 2 1 ( 1.6) 1

RENAL FAILURE CHRONIC 1 ( 1.6) 1

RESPIRATORY, THORACIC AND MEDIASTINAL DISORDERS 4 ( 8.7) 4 7 (10.9) 8

ASTHMA 1 ( 1.6) 1

CHRONIC OBSTRUCTIVE PULMONARY DISEASE 1 ( 2.2) 1

COUGH 1 ( 2.2) 1 2 ( 3.1) 2

DYSPNOEA 1 ( 2.2) 1 2 ( 3.1) 2

PLEURITIC PAIN 1 ( 1.6) 1

PNEUMONIA ASPIRATION 1 ( 2.2) 1

PRODUCTIVE COUGH 1 ( 1.6) 1

UPPER RESPIRATORY TRACT CONGESTION 1 ( 1.6) 1

SKIN AND SUBCUTANEOUS TISSUE DISORDERS 5 (10.9) 7 8 (12.5) 10

BLISTER 1 ( 1.6) 1

HAIR GROWTH ABNORMAL 1 ( 1.6) 1

PRURITUS 3 ( 6.5) 5 3 ( 4.7) 4

RASH 1 ( 2.2) 1 1 ( 1.6) 1

_____________________________________________________________________________________________________________________________________

ITT population included all subjects who received at least one dose of the study medication.

Blanks represented no adverse events recorded.

The p-values was derived using Pearson''s Chi-square test.

Data Version Date: 25SEP2008 t4.1.sas executed at 24JUL2009 13:04

Fresenius Medical Care - North America 7 of 7

EPICK

Appendix B Table 1

Adverse Events prior to Day 98 Visit - ITT population

_____________________________________________________________________________________________________________________________________

_______PhosLo________ _______Placebo_______

Subject Event Subject Event

(N=46) (N=64)

System Organ Class n(%) n n(%) n

Preferred Term p-val

_____________________________________________________________________________________________________________________________________

RASH PAPULAR 1 ( 1.6) 1

SKIN DISORDER 1 ( 1.6) 1

SKIN ULCER 1 ( 2.2) 1 1 ( 1.6) 1

VASCULAR DISORDERS 2 ( 3.1) 2

HOT FLUSH 1 ( 1.6) 1

HYPOTENSION 1 ( 1.6) 1

_____________________________________________________________________________________________________________________________________

ITT population included all subjects who received at least one dose of the study medication.

Blanks represented no adverse events recorded.

The p-values was derived using Pearson''s Chi-square test.

Data Version Date: 25SEP2008 t4.1.sas executed at 24JUL2009 13:04

Fresenius Medical Care - North America 1 of 2

EPICK

Appendix B Table 2

Adverse Events Related to Study Medication prior to Day 98 Visit - ITT population

_____________________________________________________________________________________________________________________________________

_______PhosLo________ _______Placebo_______

Subject Event Subject Event

(N=46) (N=64)

System Organ Class n(%) n n(%) n

Preferred Term p-val

_____________________________________________________________________________________________________________________________________

At Least One AE 5 (10.9) 9 (14.1) 0.1632

CARDIAC DISORDERS 1 ( 1.6) 1

PALPITATIONS 1 ( 1.6) 1

GASTROINTESTINAL DISORDERS 3 ( 6.5) 4 6 ( 9.4) 10

ABDOMINAL PAIN 1 ( 1.6) 1

CONSTIPATION 2 ( 3.1) 2

DIARRHOEA 1 ( 2.2) 1 1 ( 1.6) 1

DYSPEPSIA 1 ( 1.6) 1

FLATULENCE 1 ( 1.6) 1

FREQUENT BOWEL MOVEMENTS 1 ( 2.2) 1

GASTROOESOPHAGEAL REFLUX DISEASE 1 ( 1.6) 1

NAUSEA 1 ( 2.2) 1 2 ( 3.1) 3

VOMITING 1 ( 2.2) 1

GENERAL DISORDERS AND ADMINISTRATION SITE CONDITIONS 1 ( 2.2) 1

FEELING HOT 1 ( 2.2) 1

INVESTIGATIONS 1 ( 2.2) 2

BLOOD CALCIUM INCREASED 1 ( 2.2) 2

METABOLISM AND NUTRITION DISORDERS 2 ( 4.3) 2 1 ( 1.6) 1

ANOREXIA 1 ( 2.2) 1 1 ( 1.6) 1

APPETITE DISORDER 1 ( 2.2) 1

_____________________________________________________________________________________________________________________________________

ITT population included all subjects who received at least one dose of the study medication.

Blanks represented no adverse events recorded.

The p-values was derived using Pearson''s Chi-square test.

Data Version Date: 25SEP2008 t4.2.sas executed at 24JUL2009 15:23

Fresenius Medical Care - North America 2 of 2

EPICK

Appendix B Table 2

Adverse Events Related to Study Medication prior to Day 98 Visit - ITT population

_____________________________________________________________________________________________________________________________________

_______PhosLo________ _______Placebo_______

Subject Event Subject Event

(N=46) (N=64)

System Organ Class n(%) n n(%) n

Preferred Term p-val

_____________________________________________________________________________________________________________________________________

MUSCULOSKELETAL AND CONNECTIVE TISSUE DISORDERS 1 ( 1.6) 1

PAIN IN EXTREMITY 1 ( 1.6) 1

SKIN AND SUBCUTANEOUS TISSUE DISORDERS 1 ( 1.6) 1

HAIR GROWTH ABNORMAL 1 ( 1.6) 1

VASCULAR DISORDERS 1 ( 1.6) 1

HOT FLUSH 1 ( 1.6) 1

_____________________________________________________________________________________________________________________________________

ITT population included all subjects who received at least one dose of the study medication.

Blanks represented no adverse events recorded.

The p-values was derived using Pearson''s Chi-square test.

Data Version Date: 25SEP2008 t4.2.sas executed at 24JUL2009 15:23
